# A “Council of Caregivers” and Dementia Friends Geriatrics Person‐Centered Session for First‐Year Medical Students

**DOI:** 10.1111/jgs.70045

**Published:** 2025-08-30

**Authors:** Glenda R. Westmoreland, Kathryn I. Frank, Michael Dustin Ziegler, Melinda Winnie, Emilie L. Garrison, Sarah E. Roth, Debra K. Litzelman

**Affiliations:** ^1^ Indiana University School of Medicine Indianapolis Indiana USA; ^2^ Indiana University Indianapolis Indiana USA; ^3^ CICOA‐Aging & In‐Home Solutions Indianapolis Indiana USA; ^4^ Regenstrief Institute, Inc Indianapolis Indiana USA; ^5^ Indiana University Center for Global Health Equity Indianapolis Indiana USA

**Keywords:** Council of Elders, dementia friends, medical student education

## 1 Background

Medical schools are being urged to incorporate dementia content in the foundational years of medical education providing students with a more comprehensive picture of what is required to care for adults who have dementia [1]. We developed, implemented, and evaluated a person‐centered session on dementia for first year medical students. The goal of the session was for medical students to see the range of functionality of older adults who have dementia and recognize how to appropriately interact with both older adult and caregiver. During the first 30 min of the 90‐min session medical students interviewed a Council of Caregivers (CoC) followed by a 60‐min implementation of the Dementia Friends Indiana (DFI) curriculum. The CoC is a panel of three current or former caregivers of a parent or spouse who had dementia and was adapted from our prior work with a “Council of Elders (CoE)” session [2]. The DFI portion of the session was adapted from the world‐wide DF. The overarching goal of DF training, regardless of where it is implemented, is for participants to recognize dementia is not a normal aspect of aging [3]. DFI includes a PowerPoint presentation with a video embedded in showing an interview of three older adults who, despite having dementia, have a good quality of life.

## 2 Methods

In 2021, the Indiana Geriatrics Education and Training Center (I‐GETC) at Indiana University School of Medicine (IUSM) partnered with the Central Indiana Council on Aging and in Home Solutions (CICOA), the largest Area Agency on Aging in our state, to develop a new dementia session for first‐year medical students. IUSM is the largest allopathic medical school in the United States, with nine decentralized campuses. The new dementia session was one of 12 elective offerings during a required course, The Foundations of Clinical Practice, designed for medical students to develop clinical skills and explore the impact of psychosocial factors on health and disease. Attendance for the session was in person for the largest IUSM campus, with virtual attendance by students at other campuses.

For the first part of the session voluntary CoC members recommended by CICOA were in varying stages or roles along their caregiving journey (e.g., a millennial whose parent was deceased and a spouse actively caregiving). The CoC was prepared for the session during a 1‐h training by the geriatrician who facilitated the CoC session. The geriatrician has expertise in group facilitation. During the session, students generally asked the CoC sample questions we provided to assist them in generating their own questions.

For the second part of the session, the Director of DFI from CICOA conducted the interactive DFI training as a large group activity including all medical student participants [3]. Afterward, students broke into small groups with other DFI‐trained facilitators for 10 min to ask questions and voice observations. The large group reconvened for brief closing remarks and to complete the post survey. The survey consisted of both Likert scale and open‐ended questions. It assessed whether students found value in the session, recognized the importance of a geriatrics session in their curriculum and planned to use what they learned personally or professionally. For example, one open ended question was “What did participating in this session mean to you?” Using the qualitative description method to provide low inference, rich, and accurate description, these questions were independently evaluated by two authors (G.R.W. and S.E.R.) [4].

## 3 Results

**FIGURE 1:**
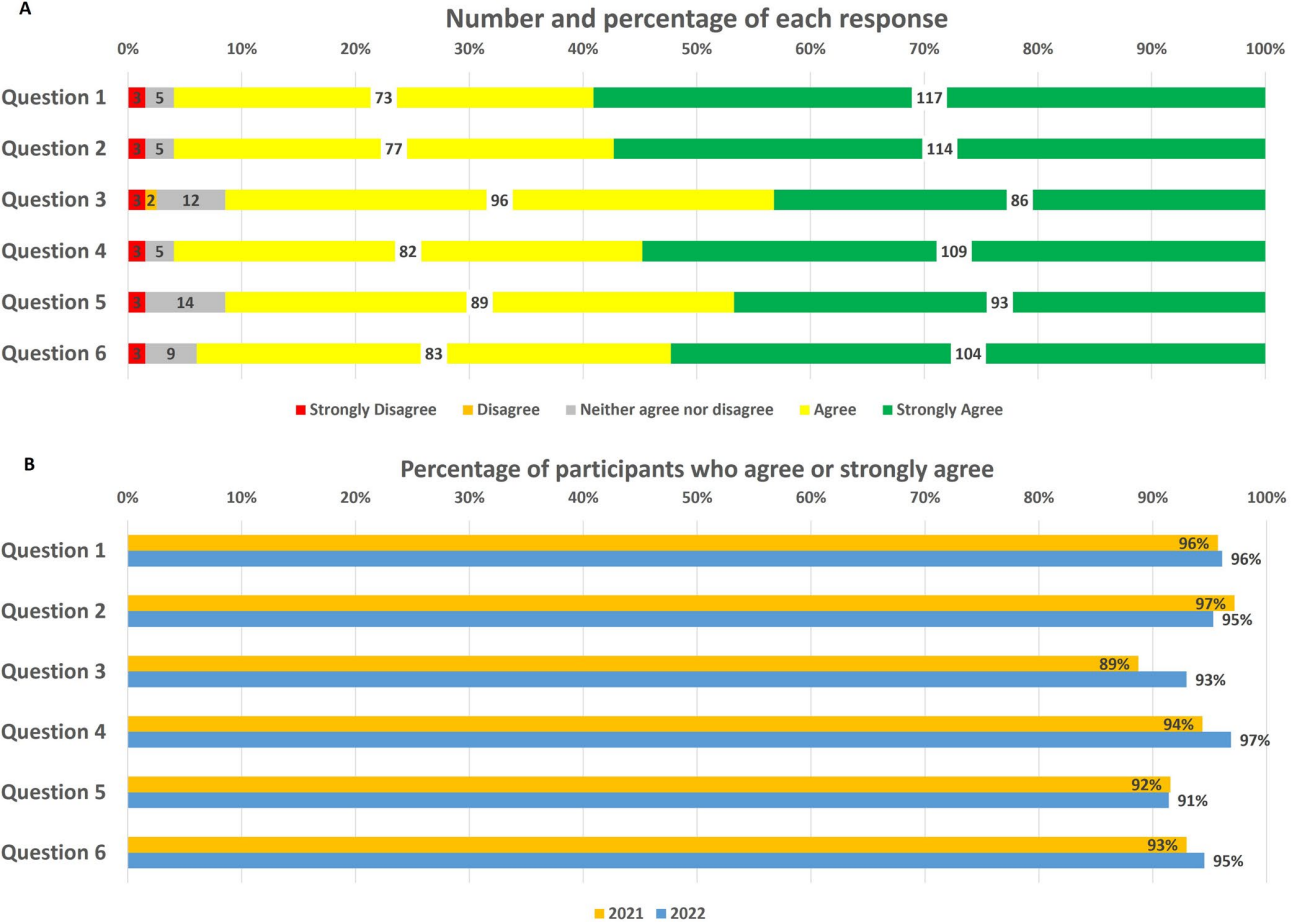
Student responses to Likert‐style questions (A) and yes/no questions (B) by percentage for both 2021 and 2022.

The dementia session has been delivered annually for two consecutive years. A total of 327 medical students attended a session. Although there are nine IUSM campuses, students from only five campuses (136 in 2021 and 191 in 2022) elected to participate in the dementia session, and among those, students from three campuses elected to complete the survey. The total survey response rate was 201 (53% (72) in 2021 and 66% (129) in 2022). Among the number of students who completed the survey from both years, 170 students (85%) saw value in requiring attendance at the dementia session. Responses to Likert‐style questions were largely favorable (see Figure 1).

When analyzing the open‐ended question, “What did participating in this session mean to you?” the authors (G.R.W. and K.I.F.) met twice to discuss, identify, and agree on major themes [4]. They achieved 97% agreement on categorizing the comments independently and, with further discussion, reached 100% agreement. Four unique themes were identified when analyzing the open‐ended question, “What did participating in this session mean to you?”: (1) Professional value of the Session for becoming a future doctor; (2) Personal connection to the Session because of a family member having dementia; (3) New perspective and/or better understanding of dementia; and (4) Challenges of caregiving and/or navigating the health care system for individuals with dementia.

## 4 Discussion

We developed, implemented, and evaluated a person‐centered dementia session for first‐year medical students for two consecutive years that included a CoC and DFI. Medical students uniformly found the session valuable and generally recommended it should be a required component of medical student education. Most medical students responded to an open‐ended prompt with comments that they found both the CoC and DFI portions to be informative. Student comments were positive about providing care as future doctors for persons with dementia and personal connections with grandparents with the disease. We will continue implementing this session and work with the course director to increase student attendance at the session.

There were limitations identified. First, students who attended the session were interested in an older adult curriculum, potentially skewing our findings toward more positive results. Second, we received less than 70% completion of our evaluations. Finally, we have not assessed the long‐term impact of the session, or was a validated tool to assess knowledge and attitudes included. In future iterations, implementing validated assessments of knowledge and attitude should be considered. The dementia session provides a promising model for medical student dementia education that can be replicated. We will use student feedback to improve the quality of this person‐centered approach, with the goal of making the session a required component of medical students' first year of training.

## Author Contributions

The author contributions can be categorized as the following. Substantial contributions to conception and design, or acquisition of data, or analysis and interpretation of data: Glenda R. Westmoreland, Kathryn I. Frank, Michael Dustin Ziegler, Melinda Winnie, Emilie L. Garrison, Sarah E. Roth, and Debra K. Litzelman. Drafting the article or revising it critically for important intellectual content: Glenda R. Westmoreland, Kathryn I. Frank, Emilie L. Garrison, Sarah E. Roth, and Debra K. Litzelman. Final approval of the version to be published: Glenda R. Westmoreland, Kathryn I. Frank, Michael Dustin Ziegler, Melinda Winnie, Emilie L. Garrison, Sarah E. Roth, and Debra K. Litzelman.

## Disclosure

This publication is supported by the Health Resources and Services Administration (HRSA) of the U.S. Department of Health and Human Services (HHS) as part of an award totaling $4,083,767 with zero percentage financed with non‐governmental sources. The contents are those of the author(s) and do not necessarily represent the official views of, nor an endorsement, by HRSA, HHS or the U.S. Government.

## Conflicts of Interest

The authors declare no conflicts of interest.

## References

[1] H. J. Roberts and J. M. Noble , “Education Research: Changing Medical Student Perceptions of Dementia,” Neurology 85, no. 8 (2015): 739–741, 10.1212/wnl.0000000000001867. 26224726 PMC4553029

[2] G. R. Westmoreland , S. R. Counsell , Y. Sennour , et al., “Improving Medical Student Attitudes Toward Older Patients Through a Council of Elders and Reflective Writing Experience,” Journal of the American Geriatrics Society 57, no. 2 (2009): 315–320. 19207146 10.1111/j.1532-5415.2008.02102.x

[3] Dementia Friends Network , Dementia Friends USA (Dementia Friends Network, 2025), dementiafriendsusa.org/.

[4] D. T. Campbell and J. C. Stanley , Experimental and Quasi‐Experimental Designs for Research (Houghton Mifflin Company, 1963).

